# Effects of plant stanol or sterol-enriched diets on lipid profiles in patients treated with statins: systematic review and meta-analysis

**DOI:** 10.1038/srep31337

**Published:** 2016-08-19

**Authors:** Shufen Han, Jun Jiao, Jiaying Xu, Diane Zimmermann, Lucas Actis-Goretta, Lei Guan, Youyou Zhao, Liqiang Qin

**Affiliations:** 1 Department of Nutrition and Food Hygiene, Jiangsu Key Laboratory of Preventive and Translational Medicine for Geriatric Diseases, School of Public Health, Soochow University, Suzhou, China; 2 Key Laboratory of Radiation Biology, School of Radiation Medicine and Protection, Soochow University, Suzhou, China; 3 Nestlé Research Centre Lausanne, Lausanne, Switzerland; 4 Nestlé Research Centre Singapore, Singapore; 5 Nestlé Research Centre Beijing, Beijing, China

## Abstract

Efficacy and safety data from trials with suitable endpoints have shown that non-statin medication in combination with a statin is a potential strategy to further reduce cardiovascular events. We aimed to evaluate the overall effect of stanol- or sterol-enriched diets on serum lipid profiles in patients treated with statins by conducting a meta-analysis of randomized controlled trials (RCTs). We used the PubMed, Cochrane library and ClinicalTrials.gov databases to search for literature published up to December 2015. Trials were included in the analysis if they were RCTs evaluating the effect of plant stanols or sterols in patients under statin therapy that reported corresponding data on serum lipid profiles. We included 15 RCTs involving a total of 500 participants. Stanol- or sterol-enriched diets in combination with statins, compared with statins alone, produced significant reductions in total cholesterol of 0.30 mmol/L (95% CI −0.36 to −0.25) and low-density lipoprotein (LDL) cholesterol of 0.30 mmol/L (95% CI −0.35 to −0.25), but not in high-density lipoprotein cholesterol or triglycerides. These results persisted in the subgroup analysis. Our meta-analysis provides further evidence that stanol- or sterol-enriched diets additionally lower total cholesterol and LDL-cholesterol levels in patients treated with statins beyond that achieved by statins alone.

Cardiovascular disease (CVD) is the leading cause of death among chronic diseases worldwide. Elevated levels of total cholesterol and low-density lipoprotein (LDL) cholesterol are important risk factors for developing CVD^1^. Extensive evidence suggests that lower levels of total and LDL-cholesterol are associated with decreased ischemic heart disease mortality^2^. Given these findings, the 2013 guidelines of the American College of Cardiology and the American Heart Association (ACC-AHA) for the treatment of cholesterol abandoned LDL targets and advocated “the lower the better” strategy^3^. In view of the robust evidence^4^, statin therapy, through inhibiting 3-hydroxy-3-methylglutaryl coenzyme A (HMG-CoA) reductase^5^, is emphasized in current US guidelines as the main treatment to reduce LDL-cholesterol. However, some patients do not reach target lipid values recommended by the National Cholesterol Education Program (NCEP) with statin monotherapy, and a long-term treatment with statin is always not been accepted in many patients due to its side effects.

Phytosterols, steroid compounds including plant stanols and sterols, present a similar structure to that of cholesterol. They are thought to decrease plasma cholesterol concentration by reducing intestinal absorption of cholesterol, upregulating hepatic expression of the LDL receptors, and decreasing production of endogenous LDL-cholesterol^6^. Studies have suggested that phytosterols may confer an additional benefit in lowering of serum lipid concentrations in patients treated with statins^7^,^8^. These compounds have therefore been recommended for patients who do not reach statin treatment targets for LDL-cholesterol and in management of mild hypercholesterolemia^9^,^10^.

Since the 1950s, numerous studies have observed the effect of phytosterols on LDL-cholesterol and several meta-analyses have evaluated their effect on serum lipid profiles^1^,^11^,^12^,^13^. Such analyses have concluded that circulating LDL-cholesterol concentration decreases with increasing phytosterol content. For example, Ras *et al*. found that LDL-cholesterol decreased as much as 12% as phytosterol administration increased up to approximately 3 g/d^13^. Similarly, a mathematical modeling approach predicted that a sterol or stanol intake of 2 g/d in combination with statins reduces LDL-cholesterol by an additional 8–9%, an effect similar to that achieved by doubling the dose of statins^14^.

Recent trials have focused on the combined effects of phytosterols and statins on lipid profiles in hypercholesterolemic patients and other patients treated with statins^7^,^15^. In a meta-analysis published in 2009, Scholle *et al*. evaluated eight randomized controlled trials (RCTs) involving hypercholesterolemic patients; they found that plant sterols or stanols combined with statins decreased the total cholesterol and LDL-cholesterol by 0.36 mmol/L and 0.34 mmol/L respectively^16^. A recent retrospective cohort study analyzed data from questionnaire responses from 3829 subjects, 43 of whom used combination treatment with statins and phytosterols (in the form of sterol- or stanol-enriched margarine) at the 5-year follow-up. Recommended margarine intake was 27 g/d. Cholesterol was reduced dose- dependently with increasing phytosterol intake (decrease of −0.0094 mmol/L for each gram of enriched margarine), with a significant reduction of 0.32 mmol/L in subjects with an intake of ≥20 g/d^17^.

An up-to-date and timely meta-analysis is important for several reasons. Previous meta-analyses have only focused on hypercholesterolemic patients, without performing comprehensive research. Since then, a large number of studies have become available, allowing the addition of subgroup analyses for important characteristics of subjects and design. We therefore performed a meta-analysis ranging from the earliest to the most recent RCTs to examine whether combined treatment of plant stanols or sterols together with statins positively affects lipid profiles compared with statins alone in treated patients.

## Results

### Study characteristics

**Figure 1:**
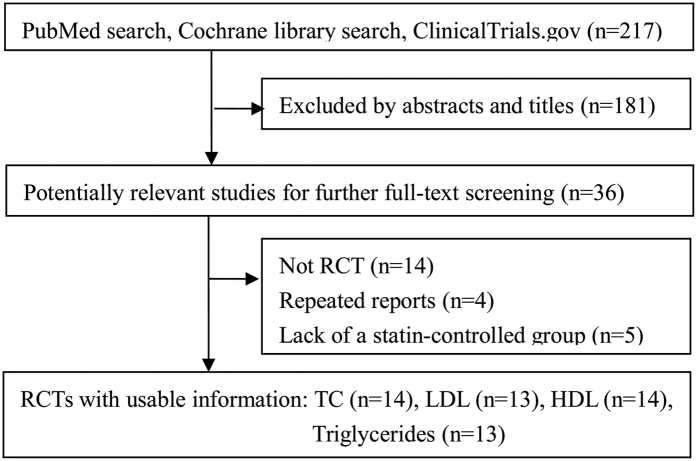
Flow Chart of study selection.

After systematic review of the literature, 14 studies, including 15 trials, satisfied the inclusion criteria for this meta-analysis (Fig. 1)^7^,^8^,^15^,^18^,^19^,^20^,^21^,^22^,^23^,^24^,^25^,^26^,^27^,^28^. The characteristics of the selected trials are presented in Tables 1 and 2. These studies were published between 1996 and 2015, and performed in the USA (*n* = 3), Netherlands (*n* = 3), Finland (*n* = 2), Spain (*n* = 1), Australia (*n* = 1), UK (*n* = 1) and Germany (*n* = 1), Portugal (*n* = 1) and Brazil (*n* = 1). Nine trials had a parallel design, and the remaining trials had a crossover design. Nine trials were double blinded, and the remaining trials were single blinded (*n* = 1), open-label (*n* = 3), or gave no information on blinding (*n* = 2). The intervention duration lasted from 4 to 85 weeks with a median of 6 weeks.

With regard to participants, twelve studies enrolled men and women, and two included men only. The number of participants in each trial varied from 8 to 141, with a sum of 382 in the parallel trials and 118 in the crossover trials. The participants in nine studies suffered from hypercholesterolemia, and the other studies involved patients with dyslipidemias, metabolic syndrome, type 1 diabetes mellitus, and impaired retinal vasculature. Not all studies provided comprehensive information of lipid index that we needed; one study did not present triglyceride data^15^ and one lacked LDL-cholesterol index^8^.

Phytosterol intake differed in these studies. Ten studies used margarine containing plant stanol or sterol ester, one study used a low-fat plant sterol-enriched fermented milk, one study administered beta-sitosterol, one study used a dried stanol/lecithin complex in tablet form, and one study received capsules containing plant sterols. Two studies combined statin therapy with plant stanols and plant sterols, respectively^15^,^23^, and one study included two trials with different cholesterol concentrations in a sitosterol-enriched diet^26^. Phytosterol dosage in the intervention group varied from 1.8 g/d to 6 g/d, with a median of 2.5 g/d. Most of the control group received no or less than 0.5 g/d phytosterols.

### Effect of phytosterols combined with statins on lipid profiles

**Figure 2:**
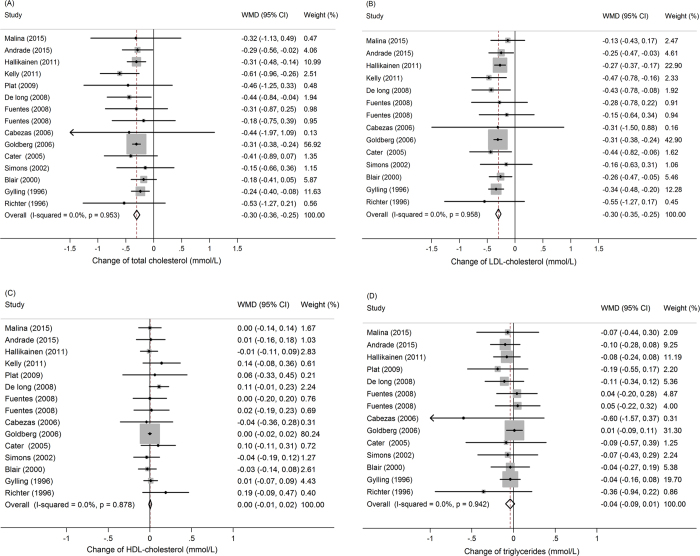
Meta-analysis of the effects of plant stanols or sterols in combination with statin on lipid profiles compared to statin control alone: changes in total cholesterol (**A**), LDL-cholesterol (**B**), HDL-cholesterol (**C**) and triglycerides (**D**). WMD, weighted mean difference.

The net changes and the corresponding 95% CIs for total cholesterol, LDL-cholesterol, HDL-cholesterol, and triglycerides are presented in Fig. 2. Compared with statins alone, combined treatment presented an average net change ranging from −0.61 mmol/L to −0.15 mmol/L for total cholesterol (Fig. 2A), with 6 of 15 trials reaching statistical significance. Similarly, the net change for LDL-cholesterol ranged from −0.55 mmol/L to −0.13 mmol/L (Fig. 2B), with 8 of 14 trials reaching statistical significance. Since no statistical heterogeneity in total cholesterol, LDL-cholesterol, HDL-cholesterol and triglyceride analyses (*I*^2^ = 0% for all), the fixed-effect model was used. After meta-analysis, with the overall effect size of combined treatment was −0.30 mmol/L (95% CI: −0.36 to −0.25) and −0.30 mmol/L (95% CI: −0.35 to −0.25) for total cholesterol and LDL-cholesterol, respectively. And the overall effect size of combined treatment was 0 (95% CI: −0.01 to 0.02) for HDL-cholesterol (Fig. 2C) and −0.04 mmol/L (95% CI: −0.09 to 0.01) for triglycerides (Fig. 2D).

### Subgroup and sensitivity analyses

Tables 3 and 4 show the results from subgroup analyses. According to subgroup analyses (Table 3), this reduction on total cholesterol was somewhat pronounced in participants with high baseline values and a low phytosterol dose of less than 3 g. This reduction on LDL-cholesterol was somewhat pronounced in participants with high baseline values, long treatment duration, and a high phytosterol dose of more than 3 g. However, the differences among all subgroups did not reach the statistical significance. The results of subgroup analyses did not reveal the effects of combined treatment on HDL-cholesterol and triglycerides (Table 4).

A sensitivity analysis was conducted by omitting one trial each in turn to yield a narrow range with minimal changes in the levels of total cholesterol (from −0.30 mmol/L to −0.31 mmol/L), LDL-cholesterol (from −0.31 mmol/L to −0.29 mmol/L) and HDL-cholesterol (from 0 mmol/L to 0.02 mmol/L). However, the overall effect size on triglycerides was −0.06 mmol/L (95% CI: −0.13 to 0.00) after excluding the trial by Goldberg *et al*.^25^ and this finding presents a different conclusion from the results of the total analysis.

Two studies, those of Kelly *et al*.^15^ and De Jong *et al*.^23^ used both plant stanols and sterols in combination with statins. In the sensitivity analyses conducted on these studies, the selected plant sterols combined with statin treatment presented an overall effect size of −0.30 mmol/L (95% CI −0.36 to −0.25) for total cholesterol, −0.30 mmol/L (95% CI −0.34 to −0.25) for LDL-cholesterol, 0 mmol/L (95% CI −0.01 to 0.02) for HDL-cholesterol and −0.04 mmol/L (95% CI −0.09 to 0.01) for triglycerides. The results from these two studies were consistent when plant stanols were used in the analyses.

### Meta-regression analyses

To minimize the likelihood of false-positive results, we carefully selected a small number of covariates, including baseline lipid level, intervention duration, and phytosterol dose. In the meta-regression analysis, none of these three covariates significantly influenced the overall effect size for total cholesterol (*P* = 0.89, 0.17, 0.95), LDL-cholesterol (*P* = 0.48, 0.22, 0.50), HDL-cholesterol (*P* = 0.43, 0.13, 0.66) and triglycerides (*P* = 0.68, 0.38, 0.88).

### Publication bias

Visual inspection of Begg funnel plot show no asymmetry in total cholesterol, LDL-cholesterol and HDL-cholesterol and some asymmetry in triglycerides (Data not shown). Further quantitative analysis showed that there was no publication bias for total cholesterol, LDL-cholesterol and HDL-cholesterol from the Begg funnel plot (*P* = 0.59, 0.78 and 0.05, respectively) or Egger regression test (*P* = 0.48, 0.88 and 0.12, respectively). However, the results for triglycerides from the Begg funnel plot (*P* = 0.03) and Egger regression test (*P* = 0.02) showed significant publication bias.

## Discussion

Our meta-analysis of 15 RCTs showed that combination treatment with statins together with phytosterols significantly decreased the levels of total cholesterol by 0.30 mmol/L and LDL-cholesterol by 0.30 mmol/L, compared with statins alone. However, combined treatment had no effect on HDL-cholesterol and triglyceride levels.

The findings have potential public health implications. Although some patients who received the combined treatment did not reach the LDL-cholesterol targets (<2.0 mmol/L)^29^, a reduction of 0.026 mmol/L (1 mg/dL) in LDL-cholesterol would be expected to decrease the relative risk for cardiovascular diseases by approximately 1%^30^. High cholesterol is a major risk factor for CVD, which can have devastating consequences and place a high potential burden of disease on patients and healthcare systems. Therefore, even a slight reduction in LDL-cholesterol may contribute to the clinical benefit of supplementary plant stanol or sterol intake. Recent strategies for cholesterol reduction have called for additional therapies beyond statins, since some patients are intolerant or do not respond adequately to statins alone^31^. Some studies have further suggested that plant stanols could be used as primary and secondary prevention with low statin doses to avoid possible adverse effects^32^. This may be due, in part, to the different mechanism of action between statins, which inhibit HMG-CoA reductase, and phytosterols, which may have a complementary effect by inhibiting cholesterol absorption.

The nutritional interest derives from the fact that phytosterols have a similar structure to cholesterol, and have the capacity to lower plasma cholesterol and LDL-cholesterol^33^. Phytosterols are specific inhibitors of intestinal cholesterol absorption and are thought to compete with cholesterol for solubilization into mixed micelles^9^, and ultimately result in an increased fecal output of cholesterol^34^,^35^. A recently published landmark study, IMPROVE-IT, is the first clinical study to show a reduction in the rate of cardiovascular events by addition of a non-statin lipid-modifying agent (ezetimibe) to statin therapy^36^. In this study, LDL-cholesterol level was 1.4 mmol/L in the simvastatin-ezetimibe group, as compared with 1.8 mmol/L in the simvastatin- monotherapy group; the effect size for LDL-cholesterol (−0.4 mmol/L) was lightly pronounced than that in our meta-analysis (−0.3 mmol/L). However, it should be interpreted with caution because of the differences between phytosterols and ezetimibe in molecular structure.

In our investigation, the observed reductions in total and LDL-cholesterol persisted through subgroup analysis. This suggests that the beneficial effects of combined treatment are probably independent and not affected by these characteristics. In addition, meta-regression analyses showed that the selected covariates, including baseline lipid level, intervention duration, and phytosterol dose, did not affect the results. Nevertheless, lifestyle modification should be noted. Although the difference after diet modification did not reach the statistical significance in the subgroup analysis, lifestyle modification is considered the cornerstone of reducing CVD risk^37^, and one of the key recommendations is the consumption of a healthy diet^38^. It remains unclear whether the effects of phytosterols on lipid profiles are dependent on lifestyle modification through decreasing dietary fat and cholesterol levels.

Although this meta-analysis was not primarily restricted by heterogeneity across the included studies, which affirmed the interpretation of our findings, certain characteristics distinguish this study from the others included in this analysis. For example, older men were selected as participants, and the baseline of serum lipid profiles were not reported. Furthermore, the overall effect size on triglycerides became significant after excluding the study by Goldberg *et al*.^25^. Some factors should be considered to explain this result. Firstly, this trial used a dried stanol/lecithin complex in tablet form rather than the stanol- or sterol-enriched margarine used in the majority of the other studies. Secondly, the dose of plant stanols (1.8 g/d) was lower than that used in other studies. Finally, statin doses were much lower in the combined treatment group than in the statin alone group.

There are a several limitations to this meta-analysis. Firstly, the sample size of individual trials was relatively small, thereby restricting the capacity of randomization to minimize the potential influences of confounding factors. Secondly, characteristics were not balanced between the treatment and control groups in some trials. For example, in one trial^8^, more participants in the combined treatment group had a higher serum baseline of total cholesterol and LDL-cholesterol than those in the statin alone group (*P* < 0.01); this disparity may obscure the effect of combined treatment on serum lipid profiles. Thirdly, the validity of our meta-analysis is dependent on the quality of the individual studies, and there were some issues with some of the trials in this regard. Specifically, allocation concealment, quality of randomization, and details of withdrawals were not always reported. Fourthly, the dose of plant sterols or plant stanols in most of the trials was 2.5 or 3 g/day, so the present meta-analysis was difficult to analyze the dose-response effect on TC and LDL-cholesterol. Finally, as with any meta-analysis, publication bias may affect the results. Although formal statistical tests did not detect evidence of publication bias, except for triglyceride results, the power of this analysis is limited because of the relatively low number of studies.

In conclusion, this meta-analysis provides evidence that phytosterol supplementation in patients treated with statins additionally decreases the levels of total cholesterol and LDL-cholesterol beyond that conferred by statins alone. Although it has been suggested that there was a lack of randomised data of the impact of phytosterol on CVD prevention^10^, enhanced consumption of phytosterol may be considered as an adjunct of statin for attainment of LDL-C goals as a function of overall CV risk can be enhanced. Well-designed RCTs must be conducted to confirm the cholesterol-lowering effect of phytosterol supplementation in patients treated with statins on CVD outcomes.

## Materials and Methods

### Data sources and study selection

We conducted a systematic literature search using the PubMed, Cochrane library and the ClinicalTrials.gov databases up to December 2015 using the following sets of search terms: (1) sterol, stanol, sitosterol, sitostanol, phytosterol, phytostanol, beta-sitosterol, beta-sitostanol, stanol ester, sterol ester and fluvastatin, cerivastatin, atorvastatin, simvastatin, pravastatin, lovastatin, rosuvastatin, statin, HMG-CoA reductase inhibitor, in combination with (2) lipids, cholesterol, high-density lipoproteins (HDL), low-density lipoproteins (LDL), and triglycerides; searches were performed with no restrictions. Only published trials reported in English were considered. Reference lists in the articles obtained from the electronic search were also manually scanned. We did not attempt to contact the authors for further information.

The criteria for the inclusion of trials were: (1) RCTs evaluating the effects of plant stanols or sterols in combination with statins; (2) trial participants treated with statins; (3) intervention duration ≥4 weeks; and, (4) reporting at least one of four suitable lipid endpoints (total cholesterol, LDL-cholesterol, HDL-cholesterol or triglycerides) including net changes and their corresponding standard deviation (SD), or available data to calculate them. Data from plant stanols were used for the main analysis when plant stanols and sterols were used, respectively, in combination with statins. When more than one follow-up time point was mentioned, the data from the longest period were used. If the study sample was found to overlap in two or more articles, only the publication with the largest sample was used.

### Data extraction and quality assessment

We recorded the study characteristics as follows: (1) first author’s name, publication year, and country of origin; (2) study design details; (3) sample size; (4) study duration; (5) source and dose of plant stanols or sterols, type and dose of statins, and diet composition; (6) participant characteristics (mean age, mean body mass index, baseline levels of lipid profiles, and health status); and, (7) net change of total cholesterol, LDL-cholesterol, HDL-cholesterol or triglycerides and their corresponding SD. We assessed the quality of individual studies by reporting the key components of the study designs instead of providing aggregate scores. The characteristics of the study design, paticipants’ characteristics and intervention duration were used as quality parameters. Two of the authors independently performed the literature search, data extraction, and bias assessment, with disagreements resolved by discussion. We attempted to follow the Preferred Reporting Items for Systematic Reviews and Meta-Analysis guidelines in the report of this meta-analysis^39^.

### Statistical analysis

For parallel trials, net changes in each index were calculated as the difference between final and baseline values in intervention and control groups, respectively. For crossover trials, net changes were calculated as the differences in mean values at the end between the intervention and control groups. Studies with no reported SD had their values imputed from standard errors, confidence interval (CI) or P values using a standard formula for the analysis^40^.

The homogeneity of the effect size among studies was tested using the Cochran Q test at a significance level of *P* < 0.10. We also calculated the *I*^2^ statistic, a quantitative measure of inconsistency across studies^41^. An *I*^2^ value > 50% was considered to indicate substantial heterogeneity across trials. In the presence of significant heterogeneity, the random-effect model was used to calculate the overall effect size; otherwise, the fixed-effect model was acceptable. Pre-specified subgroup analysis was conducted to figure out the possible effects of study designs and participant characteristics on overall effect size. A sensitivity analysis was conducted to investigate the influence of a single study on overall effect estimate by omitting one study each while pooling the results from the remainder. Additional sensitivity analyses were conducted by using data from plant sterols instead of plant stanols when both phytosterols were used in combination treatment. Furthermore, we performed meta-regression analyses to explore possible sources of heterogeneity across studies. Potential publication bias was assessed using Begg’s funnel plots and the Egger regression test^42^. All analyses were performed using STATA version 11.0 (StataCorp., College Station, TX, USA). *P* < 0.05 was considered statistically significant, except where otherwise specified.

## Additional Information

**How to cite this article**: Han, S. *et al*. Effects of plant stanol or sterol-enriched diets on lipid profiles in patients treated with statins: systematic review and meta-analysis. *Sci. Rep*. **6**, 31337; doi: 10.1038/srep31337 (2016).

## Figures and Tables

**Table 1:** Characteristic of the trials and participants in this meta-analysis.

First author	year	Country	Study design	Sample size *	Male (%)	Age (year)	BMI (kg/m^2^)	Duration (wk)	Baseline (mmol/L)			
									TC	LDL-C	HDL-C	TG
Malina	2015	Brazil	X, O	35	23	62	30.2	4	4.32	2.60	1.33	1.50
Andrade	2015	Portugal	X, O	35	11	81	29.9	6	4.31	2.37	1.26	1.48
Hallikainen	2011	Finland	P, DB	12/1211/11	5064	18–7059.5	27.225.6	4	4.195.18	2.013.21	1.581.30	1.20NR
Kelly	2011	Netherlands	P, DB	8/11	58	61	25.8	85	5.38	3.29	1.31	NR
Plat	2009	Netherlands	P	8/1015/11	6146	60.658.2	29.426.8	9	6.555.61	4.933.41	1.161.38	2.321.78
De Jong	2008	Netherlands	P, DB	15/11	46	58.3	27.0	16	5.29	3.34	1.55	1.41
Fuentes	2008	Spain	X, DB	30	50	42	26.5	4	5.95	4.00	1.38	1.18
Fuentes	2008	Spain	X, DB	30	50	42	26.5	4	5.95	4.00	1.38	1.18
Goldberg	2006	USA	P, DB	13/13	35	59.5	27.2	6	5.36	3.25	1.32	1.99
Cabezas	2006	Netherlands	P, SB	11/9	40	48.4	26.2	6	6.88	4.84	1.14	2.16
Cater	2005	USA	X, DB	10	100	66	29.5	8	NR	2.6–3.3	NR	<2.8
Simons	2002	Australia	P, DB	37/38	47	60	27.2	4	7.54	5.25	1.40	1.98
Blair	2000	USA	P, DB	69/72	60	56	29.0	8	6.01	3.85	1.38	1.77
Gylling	1996	Finland	X, B	8	100	60.2	26.6	7	≥6.0	NR	NR	≤2.5
Richter	1996	Germany	P, O	15/15	53	45.5	26.3	12	7.69	5.70	1.31	1.59

X: cross-over; P: parallel; SB: single blind; DB; double blind; B: blind; O: open-label; NR: not reported.

^*^For parallel design, sample size is intervention group/control group.

**Table 2:** Statin dose, plant sterol/stanol dose, and diet composition of the trials and participants in this meta-analysis.

First author	Statin dose	Plant sterol/stanol			Diet composition
		Intervention group		Control group dose	
		Type and dose	Esterified form		
Malina	Atorvastatin (10 mg/d) for 4 weeks run-in period, and then stable doses of atorvastatin (40 mg/d)	Plant sterol, 2.0 g/d	No	No oral plant sterols	Reinforcing lifestyle changes
Andrade	Stable statin therapy	Plant sterol, 2.0 g/d	No	Free plant sterols	Maintaining their usual dietary pattern as well as physical activity
Hallikainen	Stable doses of atorvastatin, rosuvastatin or simvastatin	Plant stanols, 3.0 g/d	Yes	about 0.1 g/d plant sterols	Vegetable oil-based spread provided by Raisio Nutrition Ltd. 64% fat for intervention group, and 49% fat for control group
Kelly	Stably with statin	Plant sterols, 2.5 g/d Plant stanols, 2.5 g/d	Yes	Free plant stanols	Keeping the normal diet and physical exercise level, and smoking and alcohol consumption
Plat	A low-dose OTC statin (10 mg simvastatin)	Plant stanol, 2 g/d	Yes	No oral plant sterols	No change in habitual diet other than low-fat yogurt drink (234 kJ/100mL)
De Jong	Stable doses of atorvastatin, simvastatin orpravastatin	Plant sterols, 2.5 g/d Plant stanols, 2.5 g/d	Yes	No added plant stanol	No change in habitual diet other than margarine use
Fuentes	Stable doses of atorvastatin or simvastatin (40 mg/d) for at least 8 weeks prior	Sitosterol, 2.5 g/d	No	<0.5 g/d plant sterol from control diet	280–300 mg/d cholesterol, <30% fat, <10% saturated fat, 6% PUFA, 12% MUFA
Fuentes	Stable doses of atorvastatin or simvastatin (40 mg/d) for at least 8 weeks prior	Sitosterol, 2.5 g/d	No	<0.5 g/d plant sterol from control diet	150mg/d cholesterol, <30% fat, <10% saturated fat, 6% PUFA, 12% MUFA
Goldberg	Stable statin dose for at least 3months prior	Plant stanols, 1.8 g/d	No	Placebo tablet containing starch	American Heart Association Heart Healthy Diet
Cabezas	Stable doses of atorvastatin or simvastatin (80 mg/d) for at least 6 months prior	Plant stanols, 3g/d	No	No adding plant stanol	Dietary education only
Cater	Stable doses of simvastatin or atorvastatin for ≥2 months prior	Plant stanol, 3 g/d	Yes	No adding plant stanol	A diet low in saturated fat (<10% daily calories) and cholesterol (<300 mg/d)
Simons	Cerivastatin (400 ug/d)	Plant sterol, 2 g/d	Yes	Virtually no serol	American Heart Association Step I diet
Blair	Stable doses of atorvastatin, pravastatin. simvastatin or lovastatin for at least 90 days prior	Plant stanol, 3 g/d	Yes	No adding plant stanol	No change in habitual diet other than margarine use for intervention group, 24 g/d of matching canola oil-based placebo margarine with average fat content of 18 g for control group
Gylling	Pravastatin (40 mg/d)	Sitostanol, 3g/d	Yes	50.2 mg/d campesterol and 69.1mg/d sitostanol	No change in habitual diet other than margarine use
Richter	Maximally tolerated dose of lovastatin (56.5 ± 25.0 mg/d)	β-sitosterol, 6 g/d	No	No oral plant sterol	A cholesterol-lowing diet as recommended by the European Atherosclerosis Society

**Table 3:** Results of subgroup analyses according to trial and participant characteristics for total cholesterol and LDL-cholesterol.

	Group	No	Net change (95% CI)	*P*-heterogeneity	*I*^*2*^ (%)
Total cholesterol					
	Total	15	−0.30 (−0.36, −0.25)	0.953	0
Baseline	≥6 mmol/L	6	−0.35 (−0.46, −0.23)	0.872	0
	<6 mmol/L	8	−0.29 (−0.35, −0.23)	0.789	0
Duration	≥7 wk	7	−0.30 (−0.41, −0.19)	0.439	0
	<6 wk	8	−0.31 (−0.37, −0.24)	0.999	0
Stanol or sterol dose	≥3 g	6	−0.29 (−0.35, −0.24)	0.816	0
	<3 g	9	−0.37 (−0.52, −0.21)	0.897	0
Diet modification	yes	8	−0.31 (−0.37, −0.25)	0.990	0
	no	7	−0.29 (−0.39, −0.18)	0.531	0
Intervention	sterol only	8	−0.34 (−0.49, −0.19)	0.931	0
	stanol only	9	−0.31 (−0.36, −0.25)	0.697	0
Study design	parallel	9	−0.31 (−0.37, −0.25)	0.715	0
	cross-over	6	−0.26 (−0.39, −0.14)	0.988	0
LDL-cholesterol					
	Total	14	−0.30 (−0.35, −0.25)	0.958	0
Baseline	≥3.5 mmol/L	6	−0.30 (−0.37, −0.23)	0.925	0
	<3.5 mmol/L	6	−0.28 (−0.36, −0.20)	0.673	0
Duration	≥7 wk	6	−0.35 (−0.45, −0.25)	0.843	0
	<6 wk	8	−0.28 (−0.34, −0.23)	0.949	0
Stanol or sterol dose	≥3 g	6	−0.30 (−0.36, −0.25)	0.868	0
	<3 g	8	−0.28 (−0.40, −0.15)	0.818	0
Diet modification	yes	7	−0.31 (−0.40, −0.22)	0.745	0
	no	7	−0.30 (−0.35, −0.24)	0.909	0
Intervention	sterol only	8	−0.27 (−0.39, −0.14)	0.868	0
	stanol only	8	−0.31 (−0.36, −0.26)	0.890	0
Study design	parallel	8	−0.30 (−0.36, −0.25)	0.881	0
	cross-over	6	−0.30 (−0.40, −0.20)	0.761	0

**Table 4:** Results of subgroup analyses according to trial and participant characteristics for HDL-cholesterol and triglycerides.

	Group	No	Net change (95% CI)	*P*-heterogeneity	*I*^*2*^ (%)
HDL-cholesterol					
	Total	15	0.00 (−0.01, 0.02)	0.878	0
Baseline	≥1.35 mmol/L	6	0.01 (−0.04, 0.06)	0.572	0
	<1.35 mmol/L	7	0.00 (−0.02, 0.02)	0.753	0
Duration	≥7 wk	7	0.04 (−0.01, 0.09)	0.460	0
	<6 wk	8	0.00 (−0.02, 0.02)	1.000	0
Stanol or sterol dose	≥3 g	6	0.00 (−0.02, 0.02)	0.687	0
	<3 g	9	0.03 (−0.02, −0.09)	0.859	0
Diet modification	yes	8	0.00 (−0.02, 0.02)	0.876	0
	no	7	0.02 (−0.03, 0.07)	0.617	0
Intervention	sterol only	8	0.04 (−0.02, 0.09)	0.776	0
	stanol only	9	0.00 (−0.01, 0.02)	0.631	0
Study design	parallel	9	0.00 (−0.02, 0.02)	0.503	0
	cross-over	6	0.02 (−0.04, 0.07)	0.981	0
Triglycerides					
	Total	14	−0.04 (−0.09, 0.01)	0.942	0
Baseline	≥1.7 mmol/L	5	−0.02 (−0.10, 0.07)	0.610	0
	<1.7 mmol/L	7	−0.07 (−0.15, 0.02)	0.842	0
Duration	≥7 wk	6	−0.07(−0.16, 0.02)	0.876	0
	<6 wk	8	−0.02 (−0.09, 0.04)	0.814	0
Stanol or sterol dose	≥3 g	6	−0.03 (−0.09, 0.04)	0.796	0
	<3 g	8	−0.07 (−0.16, 0.03)	0.852	0
Diet modification	yes	8	−0.02 (−0.09, 0.05)	0.812	0
	no	6	−0.07 (−0.15, 0.02)	0.892	0
Intervention	sterol only	7	−0.06 (−0.17, 0.04)	0.799	0
	stanol only	8	−0.04 (−0.10, 0.02)	0.824	0
Study design	parallel	8	−0.04 (−0.11, 0.03)	0.689	0
	cross-over	6	−0.04 (−0.12, 0.05)	0.930	0
